# A New Contraceptive Diaphragm in Niamey, Niger: A Mixed Methods Study on Acceptability, Use, and Programmatic Considerations

**DOI:** 10.9745/GHSP-D-21-00532

**Published:** 2022-02-28

**Authors:** Ashley Jackson, Alexandra Angel, Abdoul-Razak Mahamadou Bagourmé, Moumouni Boubacar, Aminata Maazou, Harou Issoufa, Paul Bouanchaud

**Affiliations:** a Population Services International, Washington, DC, USA.; b Population Services Internationa/Niger, Niamey, Niger.; c Ministry of Public Health, Population, and Social Affairs of Niger, Niamey, Niger.

## Abstract

**Introduction:**

Diaphragms have never been widely available in West Africa, a region with very low contraceptive use due in part to health system constraints and low demand for other modern methods of contraception. Research has demonstrated the single-size Caya contoured diaphragm to be a safe and effective barrier method of nonhormonal, self-care contraception, offering features that improve upon the design of traditional diaphragms. No studies have measured continuation of Caya. In Niamey, Niger, this pilot introduction study explored Caya acceptability, use, and programmatic considerations.

**Methods:**

Women in Niger participated in surveys (n=150) and in-depth interviews (n=25) 6 months after adopting Caya. In-depth interviews were also conducted with men in the community (n=15) and family planning providers (n=15). We conducted descriptive analysis of the percentage of adopters who continued to use the method at 6 months and women’s reasons for discontinuation among women who completed the 6-month follow-up survey. We used deductive thematic analysis of in-depth interview data from women, men, and providers to understand program elements and other factors associated with acceptability.

**Results:**

Six months after initiation, 76.7% (95% confidence interval=0.70, 0.83) of 150 survey respondents reported continued use of Caya. Top reasons for use included: Caya causes no side effects for most users, works on demand, and is reusable for up to 2 years. Roughly one-quarter of women interviewed reported inconsistent use. Some men supported their partner’s use; in other cases, women used Caya without informing or involving their partners. Providers described Caya as compatible with the needs of many women and credited pelvic models with enabling effective counseling.

**Discussion:**

The Caya diaphragm has the potential to be a valuable addition to the range of contraceptive options, especially in settings with high levels of opposition to other modern methods.

## Abstract (precis)

Through a pilot introduction in Niamey, Niger, we found that expanding method options to include the Caya diaphragm, a new self-care contraceptive product without side effects for most users, may address some of the challenges that contribute to very low contraceptive use.

## Teaser

**Key Findings:**

- Community health workers and facility-based providers in the public and private sectors successfully added the Caya diaphragm to their offering and found this new self-care contraceptive method of interest to many clients, including women who had never used modern contraception.
- Women and men described a unique value proposition for Caya: the product causes no side effects for most users, works on demand, and is reusable for up to 2 years.
- Most women who adopted Caya continued to use it after 6 months. The top reasons for discontinuation were the desire for pregnancy and concerns about effectiveness of the method.

**Key Implications:**

- In settings where health systems are overstretched and opposition to other methods of contraception is high, national stakeholders and their partners should consider adding the Caya diaphragm to the range of contraceptive options offered by community health workers and facility-based family planning providers. Introduction efforts should begin on a small scale with close monitoring to determine whether to further expand access.

Résumé en français à la fin de l’article.

## INTRODUCTION

A wide range of contraceptive method choices is needed to meet the diverse needs and preferences of individuals in a population. The introduction of new contraceptive products has the potential to increase overall contraceptive use if the new product aligns with health system realities and the preferences of consumers whose needs are unmet or poorly met by other products. In the past, modern contraceptive use has typically increased when a new method became available to at least half of the population in a country.^1^ However, the results of contraceptive product introductions have greatly varied by country and contraceptive method.^2^ In countries with fewer contraceptive options, the addition of a new method is associated with greater increases in overall contraceptive use.^1^ While some methods have seen little uptake after introduction, the surge in use of contraceptive implants in sub-Saharan Africa over the past decade is an example of how a contraceptive market can transform as a result of concerted action and investment in shaping the market for a method compatible with local systems and preferences.^3^ Pilot introduction research such as the present study is needed to inform investment and policy decisions about which new contraceptive products to make available and where.

Countries in West Africa, including Niger, have never had widespread access to diaphragms of any kind, including the newly developed Caya contoured diaphragm from Medintim. Known as the SILCS diaphragm during its development, Caya is a thin contraceptive cup that fits over the cervix to prevent sperm from entering the uterus. This woman-controlled, nonhormonal product is reusable for up to 2 years. Unlike past diaphragms, Caya comes in a single size that fits most women, simplifying procurement and service delivery. Through a user-centered process, PATH designed Caya to be easier to use than traditional diaphragms, more comfortable for the user, and accessible without a fitting by a provider.^4^ The Caya diaphragm is indicated for use with a contraceptive gel such as Caya gel (which contains lactic acid and cellulose) or a spermicide. Although Caya is marketed in the United States, Australia, much of Europe, and several other countries, the product is not yet available in any Asian or sub-Saharan African country other than Nigeria, where private sector sales of Caya began in 2020.

**Figure:**
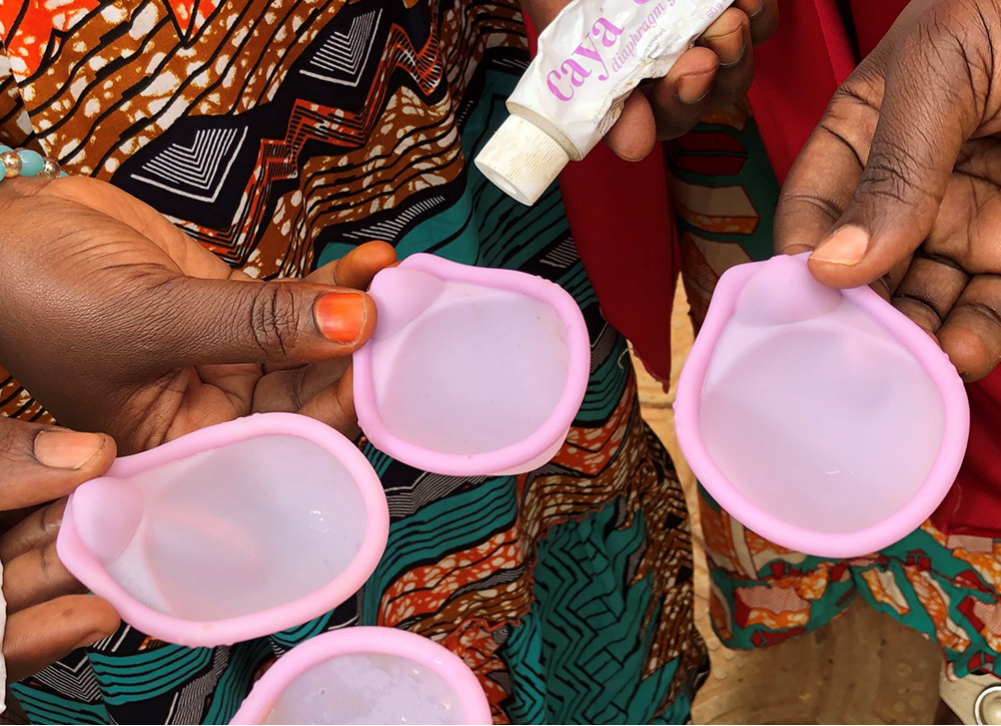
Caya diaphragm samples in Niamey, Niger. ©2019 Alexandra Angel/Population Services International.

Studies in multiple countries have demonstrated that Caya is safe, effective, and acceptable to many women, including adolescents, and their male partners.^5^^–^^10^ However, Caya acceptability research in Africa has been limited to 2 Southern Africa countries (South Africa^7^^,^^8^ and Zimbabwe^9^), with no studies to date in West Africa. Research in 2 West African countries, Nigeria and Senegal, suggested that many women found a different vaginal contraceptive product, the progesterone vaginal ring, to be acceptable.^11^ A literature gap remains about the acceptability of diaphragms in general, and Caya in particular, in West Africa, where there is low use of other vaginally inserted products such as tampons and female condoms.

Little is known about diaphragm continuation. In 2000, Stover, Bertrand, and Shelton assigned traditional diaphragms 1 couple-year of protection (CYP) per device based on “no empirical data available” on how long women typically use a diaphragm.^12^ Only 4 published studies have ever measured diaphragm continuation: 3 studies with populations outside of sub-Saharan Africa and 1 with female sex workers in Kenya in a clinical research context.^5^^,^^13^^–^^15^ Only 1 of these studies, a clinical trial in the United States, examined continuation of the Caya diaphragm specifically and the analysis did not distinguish between women who discontinued because of a desire for pregnancy and women who discontinued for other reasons.^5^ Measurement of discontinuation for any reason is needed for some purposes, such as development of a CYP factor for the method and cost-effectiveness analyses. These results can also be more easily compared to the literature on other methods. Measurement of discontinuation while in need of contraception is more relevant for other purposes, such as understanding method-related challenges including dissatisfaction and access barriers. The need to close the continuation research gap is urgent now that Caya is available and potentially well-suited to family planning (FP) programs in low-resource settings.

Because little is known about diaphragm continuation, there is an urgent need to close the continuation research gap now that Caya is available and potentially well-suited to FP programs in low-resource settings.

This study assessed Caya acceptability and use among women in Niamey, Niger. The program implementers selected this study site because Niger has one of the lowest rates of contraceptive use in the world (16% in 2018).^16^ Method/health-related concerns and perceived lack of risk of pregnancy are among the top reasons for nonuse of contraception among women in Niamey with unmet need.^17^ This study explored whether introducing Caya through public and private sector channels, including community-based distribution, would be feasible and align with the contraceptive preferences and needs of Nigerien women.

## EXPANDING EFFECTIVE CONTRACEPTIVE OPTIONS PROJECT

From June 2019 to December 2021, the Expanding Effective Contraceptive Options (EECO) project led a pilot introduction of Caya in Niamey, Niger, in close collaboration with the Ministry of Public Health, Population, and Social Affairs (MOH) of Niger. EECO supported community health workers (CHWs), FP providers in a private health clinic franchise, and FP providers in public health centers to add Caya to the range of voluntary contraceptive options they already offered. To be eligible to participate in this pilot introduction, public facilities and private clinics needed to offer, at a minimum, male condoms, oral contraceptive pills (OCPs), injectable contraceptives, implants, copper intrauterine devices, and counseling on the Lactational Amenorrhea Method, even if these methods were not always in stock.

The MOH and EECO jointly delivered training, continuing education, and supportive supervision to providers. During training, providers received their own Caya samples to take home and use themselves if they wished. Some providers chose to try the product with their partners and voluntarily shared their positive experiences with the new method with their fellow trainees. EECO also provided Caya diaphragm and gel supplies and counseling support materials. Facility-based providers and CHWs used the same educational materials during counseling: a case with samples of all available methods, posters with pictorial instructions for Caya use, and pelvic models to demonstrate diaphragm use and facilitate practice by clients.

CHWs raised community awareness of this new method within the context of informed choice through interpersonal communication (e.g., talks for groups of women in health center waiting rooms, meetings with community and religious leaders, door-to-door communication). Female CHWs reached women with interpersonal communication while a male CHW reached groups of men. All CHWs were trained and able to sell Caya directly to clients in the community or could refer clients to a public sector facility if the client preferred.

**Figure:**
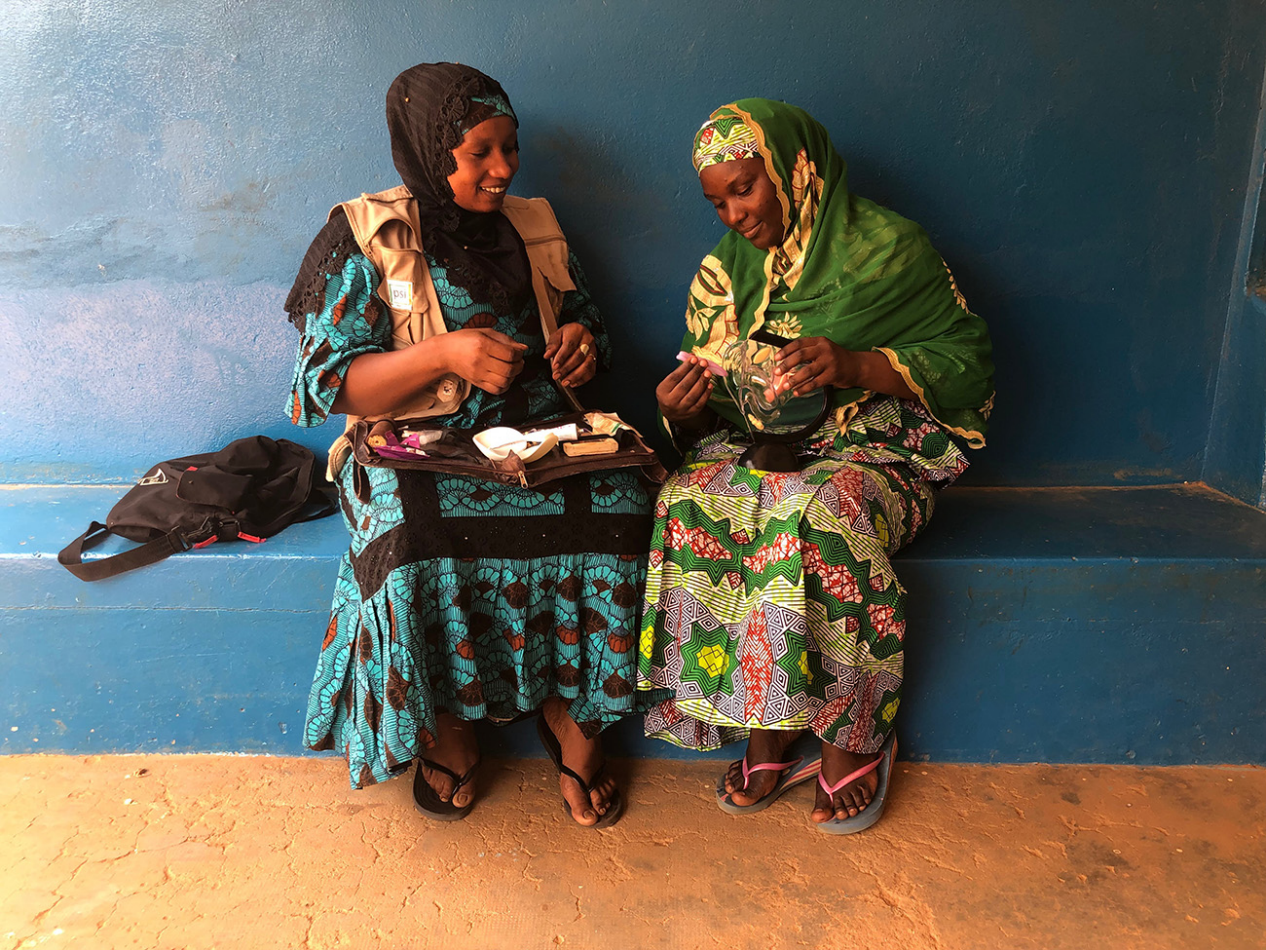
A community health worker in Niamey, Niger, counsels a woman on the Caya diaphragm. ©2019 Alexandra Angel/Population Services International.

Clients who accessed Caya through the public sector received the Caya diaphragm and gel for free, per national guidelines for the public sector in Niger. Clients who accessed Caya through CHWs or private clinics paid subsidized prices of 500 CFA ($US0.90) for a diaphragm and gel kit and 500 FCFA ($US0.90) for Caya gel resupply (each 60-milliliter gel tube lasts for about 16 uses). In comparison, the procurement prices offered by the manufacturer to this project were $US5.66 per Caya diaphragm and $US3.76 per tube of Caya gel in 2021.

More than 1,000 women adopted Caya within the first 15 months of service delivery in Niger. At the end of the pilot introduction in December 2021, providers continued to offer resupplies of Caya gel, but few had remaining stock of Caya diaphragms. From October to December 2021, CHWs reached out to diaphragm users to provide counseling on alternative method options once gel resupplies are exhausted or 2 years have elapsed since the client began using her diaphragm. As of December 2021, the MOH, donors, and implementing partners were in discussion about the possibility of scaling up access to Caya.

During the pilot project, more than 1,000 women adopted Caya within the first 15 months of service delivery in Niger.

## METHODS

This mixed methods study combined a quantitative user survey and qualitative in-depth interviews (IDIs) to assess continued use and acceptability, as well as user/provider experience, male partner involvement, and factors that enabled/impeded use. Female adopters, potential male partners, and Caya providers were included in the study populations for in-person IDIs, which included a mix of open- and closed-ended questions. The short, quantitative surveys were conducted by telephone and only with female Caya adopters.

The study’s quantitative survey objectives were to determine the proportion of adopters who continued to use the method at 6 months and to understand the reasons for continued use or discontinuation of Caya during the 6-month period.

Objectives pertaining to the IDIs were to understand clients’ experiences with Caya, including why and how they use it; understand the role of male partners in the choice and use of the method; and identify factors that enabled or impeded use.

Women aged 18–49 years in Niamey, Niger, who selected Caya as their contraceptive method 6 months before data collection were eligible to participate in the survey and IDIs, regardless of whether they continued to use the method or had ever used it. Men were eligible to participate in IDIs 1 month after they attended an FP education session that included information on Caya; men were eligible to participate in IDIs regardless of whether they and their partner were Caya users. Providers were eligible to participate in IDIs 6 months after receiving the training and supplies needed to add Caya to their voluntary FP offering.

The user survey sample size calculation was powered on the percentage of adopters who would continue to use the method at follow-up, the primary outcome. Based on previous studies of continuation with short-acting and barrier methods of contraception, 50% was used as a conservative reference for expected percentage of adopters who would continue use at 6 months.^18^ Using this baseline reference, the intended sample size at endline was 150 survey respondents. Descriptive analysis was also used to explore women’s reasons for discontinuation. After adopting Caya, a total of 255 women consented to be contacted 6 months later for participation in the survey and 5 women declined to be contacted about the survey. Data collectors stopped enrolling and contacting women after receiving 150 responses.

Insights on acceptability were derived from deductive thematic analysis of IDI data from 25 women; 15 men; and 15 providers, which included 5 CHWs, 3 providers in private clinics, and 7 providers in public health centers. These 15 providers represented all of those who were trained to offer Caya in Niamey at the time of data collection, which took place from December 2019 to September 2020. Fifteen women participated in both the IDI and the quantitative survey.

### Ethics Approval

The Research Ethics Board of Population Services International and the local institutional review board Comité National d’Ethique pour la Recherche en Santé (CNERS) approved the study. All participants were screened into the study according to eligibility criteria and provided informed consent.

## RESULTS

### Percentage of Adopters Continuing to Use the Method: Quantitative Analysis

Women in the 6-month follow-up survey were asked if they were still using the Caya diaphragm. For this primary analysis, women were classified as discontinuing the method if they reported they had stopped using Caya for any reason, including desire to become pregnant. The proportion of women who reported continuing to use Caya at 6 months was 115 of 150 (76.7%; 95% confidence interval [CI]=0.70, 0.83). A second analysis was done excluding the 13 women who discontinued because they desired a pregnancy; with these women excluded, the proportion of women who reported continuing to use Caya at 6 months was 115 of 137 (83.9%; 95% CI= 0.78, 0.90).

The 2 most common reasons given for discontinuing were the desire for pregnancy (13 of 35 women who discontinued) and the desire for a more effective method (6 of 35 women who discontinued). Of the 150 participants included in the continuation analysis, 1 stopped using the method because she became pregnant after using Caya intermittently. Table 1 presents the reasons for discontinuing use of Caya by the number and percentage of discontinuers who provided each reason. This was fielded as a single response question; respondents could give only their primary reason for discontinuing use.

The 2 most common reasons given for discontinuing were the desire for pregnancy and the desire for a more effective method.

**TABLE 1.** Reasons for Discontinuing Use of Caya Diaphragm by 6 Months, Among Those Who Discontinued (n=35)

**Reason for Discontinuation**	**No. (%), n=35**
Desire for pregnancy	13 (37)
Wanted a more effective method	6 (17)
Opposition of male partner	3 (9)
Infrequent sex/divorce/travel	3 (9)
Not able to insert it/insufficient knowledge of the method	2 (6)
Afraid to use it	2 (6)
Inconvenient to use	1 (3)
Became pregnant	1 (3)
Other	4 (11)

### Perspectives of Caya Adopters, Men, and Providers: Qualitative Analysis

Of the 25 women who participated in IDIs, just more than half received their Caya diaphragm from a CHW; the remaining participants were split evenly between those having accessed Caya through public and private health facilities. This split mirrored the trend of Caya uptake during the first 6 months of the pilot introduction. The mean age of Caya adopters who completed IDIs was 30 years, all were married, and only 2 wanted no more children (N=25, Table 2). Demographic data were only gathered from female Caya adopters who participated in IDIs.

**TABLE 2.** Demographic Information for Caya Diaphragm Adopters Who Participated in In-Depth Interviews (N=25)

**Participant Characteristics**	**No., N=25**
Age range, years	
18–19	1
20–24	5
25–29	6
30–34	7
35–39	1
40–44	5
Marital status	
Married	25
Not married	0
Wants more children in future	
Yes	23
No	2

IDIs with Caya adopters, men, and providers shed light on 7 themes: (1) method use and satisfaction, (2) past contraceptive use, (3) intermittent use, (4) nonuse and discontinuation, (5) use of the accompanying Caya gel, (6) male engagement and discreet use, and (7) programmatic considerations.

#### Reasons for Use

Providers described Caya as compatible with the needs of many women. Several said Caya is ideal for women who cannot use hormonal methods due to contraindications. Women commonly reported having chosen the Caya because it contains no hormones and causes no side effects. Adopters expressed worries that other methods could carry health risks, such as high blood pressure, and/or expressed wanting to avoid the menstrual bleeding changes induced by some contraceptives.

*It is not harmful and it does not cause hypertension or disruption of periods*. —Caya adopter, aged 34 years

Many Caya adopters recounted personal, unsatisfactory experiences with other modern methods including OCPs, injectables, and implants.

*I saw that it's easier than pills. Pills give me a lot of problems.* —Caya adopter, aged 24 years

*[With] the pill that I often take, in a single month I can see my period twice. That's why it's not easy and I changed [methods].* —Caya adopter, aged 26 years

In addition, women frequently cited Caya’s on-demand nature as a reason why they chose this method over others. Adopters described the method as “easy” to use in contrast with OCPs, which require daily action.

*People won’t forget it, unlike medicine [OCPs] that we can forget to take.* —Caya adopter, aged 40 years

A number of Caya users noted that they have infrequent sex, typically due to prolonged periods of geographic separation from partners. For instance, a woman said her husband often travels for work for 3 months at a time. Some of these female Caya users who have infrequent sex saw other methods as incompatible with their lifestyles.

*Because I am often away from home —I travel for work for 2-week periods —you see, that’s why I don’t use pills*. —Caya adopter, aged 30 years

Providers reported that they predict Caya will gain success in Niger because, unlike condoms, it is reusable for 2 years and it does not interrupt sexual intercourse when inserted in advance. Some men and women also compared this new method to condoms, saying they preferred Caya. A male respondent, whose partner is a Caya user, reported that he is not able to feel the diaphragm during sex. A woman explained that she views Caya as more convenient than condoms because it is reusable.

*You have it there without having to leave the house.* —Caya adopter, aged 27 years

Providers reported that they predict Caya will gain success in Niger because, unlike condoms, it is reusable for 2 years and it does not interrupt sexual intercourse when inserted in advance.

Another reason women cited for choosing Caya was that they heard about it through friends or trusted community leaders, such as at Koranic school.

Nearly all (24 of 25) women expressed being satisfied or highly satisfied with Caya. In particular, women reported satisfaction with the method’s ability to prevent pregnancy without disrupting menstrual cycles. Providers voiced surprise by the high client demand for and satisfaction with Caya.

*At the beginning, even I was not convinced that women would take up this method, but throughout the sensitization efforts, many women have adopted this method and they’re satisfied with it.* —CHW

Most (22 of 25) of the women reported sharing positive Caya experiences with friends or family; 10 of 15 men also said they imparted their knowledge of the method with others. Several women reported that others adopted Caya after hearing about it from them.

#### Past Contraceptive Use

When asked about past contraceptive use, women typically responded that they had tried and been unsatisfied with hormonal methods. Four of the 25 women interviewed had never used any modern contraception before Caya. Most women were not using any modern method the day before adopting Caya, while 11 users switched directly from another method, most commonly OCPs. Some switched from traditional methods. For example, a woman reported that before adopting Caya she prevented pregnancy with amulets.

*Amulets worn on my hip and religious invocations.* —Caya adopter, aged 34 years

#### Intermittent Use

Nearly all (24 of 25) Caya adopters who participated in IDIs reported that they continued to use Caya at 6 months postadoption. While most adopters (18 of 25) reported they “always” use the method, a sizable minority (6 of 25) use the method “sometimes,” including 3 who had not used it at last sex. The 1 female IDI participant who discontinued reported she was pregnant; this was the same individual who participated in the quantitative survey and became pregnant after inconsistent use.

Women listed a range of reasons for intermittent use of Caya, such as fluctuations in their desire for pregnancy, feeling “too lazy” to use the method sometimes, and not wanting to keep it inserted 6 hours after sex. Four women reported they alternated or combined use of Caya with another modern method, primarily OCPs. Some said they combined methods to increase protection from pregnancy, while others alternated OCP and Caya based on how they were feeling.

#### Reasons for Nonuse and Discontinuation

When Caya adopters were asked why others might not choose the method, a common response was that nonusers simply did not know about Caya yet. Women listed other possible reasons, including: the misconception that the product would enter the uterus, the length of time users must wait after use before removing Caya, lack of partner support, and because the product might seem too large. For example, an adopter reported:

*I have a friend who says the diaphragm is too big for her. But I explained that if she uses gel, it will be okay.* —Caya adopter, aged 30 years

Providers reported that some clients do not choose Caya because they worry the requirement to leave Caya inserted for 6 hours after sex conflicts with the Islamic religious practice of cleansing one’s body before prayer. Some men voiced similar concerns about women leaving Caya inserted during prayer. In IDIs, women who adopted Caya did not raise this issue as a concern for themselves. Providers added that the size of the Caya gel tube was unattractive to some women who worried others might see the tubes in their pockets, drawing attention to their contraceptive use.

A few providers reported they had clients discontinue use because they found the method uncomfortable, too large, or “irritating”; they were uncomfortable with the insertion process; their husbands said they could feel Caya during sex; and/or their husband refused to use it. During the 2 years and 3 months of this pilot introduction, Caya users reported no serious adverse events.

#### Use of Accompanying Gel

All 25 women interviewed reported they used gel with their diaphragm, with 24 using Caya gel and 1 using a water-based lubricant. Nearly all (24 of 25) women used a gel the last time they used the Caya diaphragm. Women reported being able to access the amount of Caya gel they needed. Providers described hearing both positive and negative comments from women concerning Caya gel; positive comments—such as appreciating the gel’s vanilla scent—were more common, though negative comments were also reported, with a provider sharing that some of her clients had found the gel irritating. Among the Caya users interviewed, several appreciated the lubricating effect of Caya gel. In reference to its lubricating quality, a woman stated:

*It doesn’t annoy us. He [my husband] loves it.* —Caya adopter, aged 26 years

Providers reported that potential clients commonly ask if Caya gel can affect a woman’s health and future fertility. A CHW reported that women also ask if they can use Caya gel alone for contraceptive purposes. (Caya gel does not affect future fertility and does not prevent pregnancy on its own.)

#### Male Engagement and Discreet Use

The majority of adopters (18 of 25) reported that their male partner played a role in the decision to use FP. Most of these women also said their partner helped make the specific choice to use Caya. One young adopter described her partner’s response to her interest in Caya:

*He said it is good. You know, nowadays no one likes closely spaced pregnancies.* —Caya adopter, aged 20 years

Women reported partner support in the form of encouragement to seek contraception and/or financial contribution to access it. Of the 15 men interviewed, 2 said their partners adopted Caya with their financial support. In 1 of these cases, the man reported he helps with insertion and removal.

Most adopters (16 of 25) believed their husbands were satisfied with Caya. Two adopters reported their husbands were not satisfied or disapproved of FP use in general. The remaining 7 women said their husband was unaware of their Caya use or had not expressed either satisfaction or dissatisfaction with the method. Similarly, 4 of 15 providers noted they know of clients who use the method without their husband’s knowledge.

Most men who participated in an education session had a positive opinion of Caya, describing it as important, useful, practical, safe, and easy to use. Nearly all (12 of 15) men said they were interested in trying it and some (4 of 15) believed they would prefer Caya over other methods, with 1 noting that this is because it is without side effects. Yet, some men did express concerns: a minority of men interviewed (3 of 15) worried that Caya could enable women and girls to commit adultery.

#### Programmatic Considerations

Most Caya adopters learned about the new method from a CHW. Other sources of information included facility-based providers, friends, relatives, and neighbors. Almost all (24 of 25) women would prefer to ask CHWs, rather than facility-based providers, their questions about the method. Women explained that they feel more comfortable confiding in CHWs and asking them “trivial” or “intimate” questions. Adopters indicated a high degree of familiarity with their CHWs, with most noting that they spoke with CHWs multiple times before and after adopting the method.

Caya adopters indicated a high degree of familiarity with their CHWs, with most noting that they spoke with CHWs multiple times before and after adopting the method.

All providers used a pelvic model to demonstrate method use and placed a high value on these demonstration tools. Most users were able to insert Caya correctly the first time they tried, but others needed up to 6 attempts to feel comfortable with insertion and removal. All women interviewed reported cleaning and storing Caya as instructed without difficulty.

## DISCUSSION

These findings suggest that—even when new and unfamiliar—Caya can appeal to women and couples in settings where contraceptive use is low. Women found Caya easy to use, many men found it acceptable, and providers saw it as compatible with many women’s needs.

Women’s stated reasons for choosing this nonhormonal method revealed concerns about the safety and side effects of hormonal contraceptives. Providers may contribute to these concerns, as some of them described Caya as ideal for women who cannot use hormonal contraception. While there are several medical conditions that make use of estrogen-containing methods unsafe, most women are in fact eligible for progestin-only contraceptives. Globally, misconceptions about the safety and reversibility of contraceptive-induced menstrual bleeding changes are common.^19^ Introducing a new product to expand method mix is important for access and choice, but it is equally important that clients have accurate information about eligibility and safety of all available methods.

The results also highlighted how few contraceptive options Nigerien women currently have or perceive having. Many women said they chose Caya because they did not want to use OCPs, suggesting that OCPs may have been the only other method they considered using. The method mix in Niamey skews toward OCPs, the method used by 58% of married women who use modern contraception.^20^ This skew may signal low availability of other methods, provider bias, lack of awareness, or misinformation about other methods.

The results also highlighted how few contraceptive options Nigerien women currently have or perceive having.

The finding that more than half of the 25 women interviewed were not using contraception before choosing Caya supports the premise that Caya could serve as an entry point for new users. That women found Caya “easy to use” is important because convenience and ease of use affect satisfaction and continuation, and because the effectiveness of self-use methods depends on a user’s correct and consistent use.

The educational resources used to train Caya providers and CHWs helped orient them to this method. Women confirmed that pelvic models aided counseling on how to use an intravaginal barrier method, and that viewing an insertion demonstration on a pelvic model inspired confidence that they too could locate their cervix and insert the product correctly. All providers also described models as valuable counseling tools. The need for educational tools such as pelvic models should be factored into efforts to expand access to this method. For CHWs who are mobile in the community, pelvic models need to be light and easily transportable. In the case of facility-based providers and CHWs alike, costs can be reduced if the same pelvic model is used for the demonstration of multiple methods (e.g., female condoms, diaphragms). In addition, pretesting of educational materials is crucial: the poster with instructions for use of Caya underwent multiple rounds of revision based on feedback from women in Niger to ensure that it was culturally appropriate and easily understood.

About 1 quarter of women surveyed reported intermittent use of Caya. This is not uncommon among user-controlled contraceptives and contributes to lower “typical use” effectiveness. With typical use, diaphragms are 83% effective, meaning 17 of every 100 women using the diaphragm are expected to become pregnant over the first year.^21^ The failure rate of this method and intermittent use explain why 1 participant became pregnant within 6 months of adopting Caya. The method’s effectiveness mattered to some who initially adopted Caya; desire for a more effective method was the second most common reason for discontinuation among those surveyed (6 of 35). Other women, such as those who had never used modern contraception before adopting Caya, prioritized other considerations over effectiveness. Some users reported that they alternated use of Caya and use of OCPs, which was not discussed in provider trainings. This practice suggests that future roll-out should include counseling for women opting to use OCPs or other methods alongside Caya to ensure women use chosen methods effectively.

Women who have infrequent sex because they or their husbands travel regularly particularly appreciated the on-demand nature of Caya because they did not have to use a method while their partner was away. For women who have infrequent sex and therefore infrequent need, the Caya diaphragm can serve as an on-demand option that is available at home and ready to use on short notice only when needed, including as a backup method during lapses in use of short-acting methods like OCPs.

Women appreciated that the Caya diaphragm could serve as an on-demand option that is available at home and ready to use on short notice only when needed.

Men’s roles in Caya use and their views of the method varied. In IDIs with female adopters, some women reported their husbands supported and enabled use. In IDIs 1 month after an information session, men shared largely positive opinions of Caya and many reported an interest in trying it. At the same time, other adopters reported their husbands were uninvolved with the decision to use Caya or were unaware the adopter was using it. Some men voiced concern that access to Caya could contribute to promiscuity; however, this hypothetical concern may have reflected their views about all contraceptive methods, not just diaphragms. While global data shows that many women conceal contraceptive use from their partner,^22^^–^^25^ significant research also confirms that in diverse settings, including in Niger, partner communication about contraception is associated with contraceptive continuation.^26^^,^^27^ The present study confirmed that women feel Caya can be used without their partner’s knowledge. However, the study also found examples of how outreach to men led to partner communication and male support for voluntary contraceptive use.

The study did not find embarrassment or stigma related to women touching their genitals as a significant barrier to Caya use, although this issue was raised by a few providers. A more common concern cited by Caya users was the 6-hour postcoital wait before diaphragm removal, a drawback that women in other diaphragm studies have also raised.^28^^,^^29^ Future clinical research could explore whether Caya could be removed sooner after sex and remain effective.

Qualitative and quantitative data from this study support the conclusion that Caya is acceptable to many women and men and has potential to be popular with those seeking nonhormonal and/or on-demand contraception. The acceptability of Caya among women who have infrequent sex is promising, especially given that infrequent sex is the second most reported reason for nonuse of existing contraceptive options among women with a need in low- and middle-income countries.^30^ Since it does not affect bleeding patterns, has no side effects for most users, and is user-controlled, Caya offers a valuable addition to the range of contraceptive options especially in settings with significant opposition to other modern methods. Further, because the method requires minimal provider interaction and is reusable for 2 years, Caya might relieve stress on overburdened health systems. The fact that lay health workers successfully offered this method suggests that Caya could be added to the limited basket of FP methods currently offered through community-based distribution.

These results can be used to inform future studies and stakeholders’ decisions related to the addition of the Caya diaphragm to FP programs in Niger and other countries. The authors recommend policy makers weigh the cost and feasibility considerations of adding the Caya diaphragm and an accompanying contraceptive gel alongside its potential positive impact on reproductive health and choice, grounding these assessments in an understanding of consumer preferences and health system realities in each context. Commercial introduction through pharmacies or partially subsidized public-private partnerships may provide sustainable avenues for introduction in some settings.

### Limitations

This pilot study had a limited implementation timeframe, which did not allow for collection of data on the percentage of women who continued to use Caya at 12 months. In addition, while this study focused on 1 method, future research could compare uptake of Caya with that of other methods at the same sites. The study design did not enable a comparative analysis of the characteristics of clients who chose Caya and those who chose other contraceptive methods; such an analysis would yield useful data to inform market segmentation for future introductions and answer questions about acceptability among women aged younger than 25 years, for example. In IDIs, open-ended questions were used to explore drawbacks of the method and future research might use these data as a starting point for more in-depth exploration of barriers to consistent use and continuation. The required brevity of the telephone survey was a constraint; for example, future research could explore characteristics that distinguish continuers from discontinuers. Following completion of IDIs, but midway through collection of survey data, the coronavirus disease (COVID-19) pandemic reached Niger. The pandemic may have influenced women’s decisions to continue or discontinue using Caya. Finally, more research is needed to investigate the feasibility of distribution and uptake in rural settings where diaphragm cleaning and accessing gel resupplies may be more challenging.

## Translation

**En Français**

**UN NOUVEAU DIAPHRAGME CONTRACEPTIF À NIAMEY, NIGER: UNE ÉTUDE DE MÉTHODES MIXTES SUR L'ACCEPTABILITÉ, L'UTILISATION ET LES CONSIDÉRATIONS PROGRAMMATIQUES**

**Résumé****Introduction:** Les diaphragmes n'ont jamais été largement disponibles en Afrique de l'Ouest, une région où l'utilisation des contraceptifs est très faible en raison en partie des contraintes du système sanitaire ainsi que la faible demande d'autres méthodes modernes de contraception. La recherche a démontré que le diaphragme Caya à taille unique est une méthode de barrière sûre et efficace de contraception non hormonale et d'autosoins, offrant des caractéristiques avec une conception améliorée par rapport aux diaphragmes traditionnels. Aucune étude n'a mesuré l’utilisation continue du Caya. À Niamey, Niger, cette étude d'introduction pilote a exploré l'acceptabilité, l'utilisation et les considérations programmatiques du Caya. **Méthodes:** Des femmes au Niger ont participé à des sondages (n=150) et à des entretiens approfondis (n=25) 6 mois après l’adoption du Caya. Des entretiens approfondis ont également été menés avec des hommes de la communauté (n=15) et des prestataires de planification familiale (n=15). Nous avons effectué une analyse descriptive du pourcentage d’adoptants qui ont continué à utiliser la méthode à 6 mois, et des raisons d'abandon parmi les femmes qui ont répondu à l'enquête de suivi à 6 mois. Nous avons utilisé une analyse thématique déductive des données d'entretiens approfondis auprès de femmes, d'hommes et de prestataires pour comprendre les éléments du programme et d'autres facteurs associés à l'acceptabilité. **Résultats:** 6 mois après l’adoption, 76,7% (intervalle de confiance à 95% = 0,70, 0,83) des 150 répondants au sondage ont déclaré qu’elles ont continué à utiliser le Caya. Les principales raisons d'utilisation: le Caya ne provoque aucun effet secondaire pour la plupart des utilisatrices; il fonctionne à la demande; et il est réutilisable jusqu'à 2 ans. Environ un quart des femmes interrogées ont signalé une utilisation irrégulière. Certains hommes ont appuyé l'utilisation du Caya par leur partenaire; dans d'autres cas, les femmes ont utilisé le Caya sans en informer ou impliquer leurs partenaires. Les prestataires ont décrit le Caya comme conforme avec les besoins de nombreuses femmes et ils ont reconnu que les modèles pelviens permettent un counseling efficace. **Discussion:** Le diaphragme Caya a le potentiel d'être un ajout précieux à la gamme d'options contraceptives, en particulier dans les contextes où les niveaux d'opposition aux autres méthodes modernes sont élevés.

**Principales Conclusions**

• Les agents de santé communautaires et les prestataires de soins des établissements du secteur public et privé ont ajouté avec succès le diaphragme Caya à leur offre et ils ont trouvé cette nouvelle méthode contraceptive d'autosoins intéressante pour de nombreuses clientes, y compris des femmes qui n'avaient jamais utilisé de contraception moderne.

• Les femmes et les hommes ont décrit une proposition de valeur unique pour Caya: le produit ne provoque aucun effet secondaire pour la plupart des utilisatrices, fonctionne à la demande et il est réutilisable jusqu'à 2 ans.

• La plupart des femmes qui ont adopté le Caya ont continué à l'utiliser après 6 mois. Les principales raisons d'arrêt étaient le désir de grossesse et les inquiétudes quant à l'efficacité de la méthode.

**Implications Clés**

• Dans les contextes où les systèmes de santé sont surchargés et où l'opposition aux autres méthodes de contraception est élevée, les parties prenantes nationales et leurs partenaires devraient envisager d'ajouter le diaphragme Caya à la gamme d'options contraceptives proposées par les agents de santé communautaires et les prestataires de planification familiale des établissements. Les efforts d'introduction devraient commencer à petite échelle avec une surveillance étroite pour déterminer s'il faut étendre davantage l'accès.

**Peer Reviewed**

**First published online:** February 18, 2022.
